# Effects and microbiota changes following oral lyophilized fecal microbiota transplantation in children with autism spectrum disorder

**DOI:** 10.3389/fped.2024.1369823

**Published:** 2024-05-09

**Authors:** Youran Li, Pei Xiao, Rong Cao, Jun Le, Qiao Xu, Fangfei Xiao, Lin Ye, Xufei Wang, Yizhong Wang, Ting Zhang

**Affiliations:** ^1^ Department of Gastroenterology, Hepatology and Nutrition, Shanghai Children’s Hospital, School of Medicine, Shanghai Jiao Tong University, Shanghai, China; ^2^ Institute of Pediatric Infection, Immunity and Critical Care Medicine, School of Medicine, Shanghai Jiao Tong University, Shanghai, China

**Keywords:** autism spectrum disorder, fecal microbiota transplantation, gut microbiota, treatment, children

## Abstract

**Background and purpose:**

Autism spectrum disorder (ASD) is a group of heterogeneous neurodevelopmental disorders that is characterized by core features in social communication impairment and restricted, repetitive sensory-motor behaviors. This study aimed to further investigate the utilization of fecal microbiota transplantation (FMT) in children with ASD, both with and without gastrointestinal (GI) symptoms, evaluate the effect of FMT and analyze the alterations in bacterial and fungal composition within the gut microbiota.

**Methods:**

A total of 38 children diagnosed with ASD participated in the study and underwent oral lyophilized FMT treatment. The dosage of the FMT treatment was determined based on a ratio of 1 g of donor stool per 1 kg of recipient body weight, with a frequency of once every 4 weeks for a total of 12 weeks. In addition, 30 healthy controls (HC) were included in the analysis. The clinical efficacy of FMT was evaluated, while the composition of fecal bacteria and fungi was determined using 16S rRNA and ITS gene sequencing methods.

**Results:**

Median age of the 38 children with ASD was 7 years. Among these children, 84.2% (32 of 38) were boys and 81.6% (31 of 38) exhibited GI symptoms, with indigestion, constipation and diarrhea being the most common symptoms. Sample collections and assessments were conducted at baseline (week 0), post-treatment (week 12) and follow-up (week 20). At the end of the follow-up phase after FMT treatment, the autism behavior checklist (ABC) scores decreased by 23% from baseline, and there was a 10% reduction in scores on the childhood autism rating scale (CARS), a 6% reduction in scores on the social responsiveness scale (SRS) and a 10% reduction in scores on the sleep disturbance scale for children (SDSC). In addition, short-term adverse events observed included vomiting and fever in 2 participants, which were self-limiting and resolved within 24 h, and no long-term adverse events were observed. Although there was no significant difference in alpha and beta diversity in children with ASD before and after FMT therapy, the FMT treatment resulted in alterations in the relative abundances of various bacterial and fungal genera in the samples of ASD patients. Comparisons between children with ASD and healthy controls (HC) revealed statistically significant differences in microbial abundance before and after FMT. *Blautia*, *Sellimonas*, *Saccharomycopsis* and *Cystobasidium* were more abundant in children with ASD than in HC, while *Dorea* were less abundant. After FMT treatment, levels of *Blautia, Sellimonas*, *Saccharomycopsis* and *Cystobasidium* decreased, while levels of *Dorea* increased. Moreover, the increased abundances of *Fusicatenibacter, Erysipelotrichaceae_UCG-003, Saccharomyces, Rhodotorula, Cutaneotrichosporon* and *Zygosaccharomyces* were negatively correlated with the scores of ASD core symptoms.

**Conclusions:**

Oral lyophilized FMT could improve GI and ASD related symptoms, as well as sleep disturbances, and alter the gut bacterial and fungal microbiota composition in children with ASD.

**Clinical Trial Registration:**

Chinese Clinical Trial Registry, ChiCTR2200055943. Registered 28 January 2022, www.chictr.org.cn.

## Introduction

Autism spectrum disorder (ASD) is a group of heterogeneous neurodevelopmental disorders that is characterized by core features in social communication impairment and restricted, repetitive sensory-motor behaviors (1). The prevalence of ASD varies across regions and different age groups, possibly due to the interplay between community awareness, service capacity and socio-demographic factors. In the United States, ASD affects about 2.3% of children aged 8-year-old and about 2.2% of adults (2). The Global Burden of Disease Study 2019 reported that the prevalence of ASD was 0.37% (370.00/100,000) and is higher in men than in women (ratio >3 : 1) (3). The global burden of ASD continues to grow and is still a main mental health concern (4). Although there is currently no curative treatment for ASD, the primary therapy is early intensive behavioral intervention, best known in the form of Applied Behaviour Analysis (2). However, no targeted medications have been developed specifically for ASD (5).

At present, ASD is a condition with unknown etiology and pathogenesis, which may be related to genetic factors, environmental factors and microbiota-gut-brain axis (1). While numerous studies have observed differences in gut microbiota between individuals with ASD and healthy controls, further research is necessary to consolidate these findings (6). Compared with the control group, Bacteroidetes, Firmicutes and Actinobacteria were more abundant in children with ASD, and the abundance of *Bacteroides, Parabacteroides, Clostridium, Faecalibacterium* and *Phascolarctobacterium* was significantly higher in children with ASD, while the abundance of *Coprococcus* and *Bifidobacterium* was lower (7). Gut microbiota affects brain development and neurobehavior through immune, neural and metabolic pathways (8). Therefore, the microbiota-gut-brain axis plays a vital role in ASD and has been considered to be a therapeutic target for ASD (9).

Fecal Microbiota Transplantation (FMT) refers to the transfer of stool from a healthy donor into the gastrointestinal tract of another patient to restore homeostasis in the recipient's intestine (10). Currently, clostridium difficile infection and recurrent clostridium difficile infection are considered to be the most appropriate indications for FMT in children. Moreover, FMT has also shown promising therapeutic effects in other pediatric diseases, including inflammatory bowel disease, irritable bowel syndrome, functional constipation, non-alcoholic fatty liver disease, ASD and diabetes (11). Although FMT has shown promise in treating various pediatric conditions, it remains under research and is not yet approved by clinical guidelines. In animal experiments, FMT derived from the healthy human gut microbiota has demonstrated significant improvements in anxiety-like and repetitive behaviors in mice with ASD and elevated serum levels of chemokines that play crucial roles in neurodevelopment and synaptic transmission within the central nervous system (12). Another study observed that FMT from naive wild-type mice effectively ameliorated memory deficits and social withdrawal in Fmr1 knockout mice. This therapeutic intervention resulted in the restoration of *Akkermansia muciniphila* level to those observed in wild-type mice, while concurrently reducing the levels of TNF-α and Iba1 in the brains of the treated mice (13). Additionally, FMT therapy has been found to repair impaired social interactions in mouse models of ASD induced by propionic acid (14). FMT treatment exhibited the ability to restore the balance of *Clostridium* species in the fecal microbiota, normalize hippocampal brain-derived neurotrophic factor expression, and increase glutathione S-transferase levels in the brain (14, 15). Overall, FMT could be a useful tool to alter the gut microbiota and improve the social withdrawal symptoms and cognitive deficits in ASD animal models, which is a promising treatment strategy for children with ASD.

It has been reported that the prevalence of gastrointestinal (GI) symptoms in autism community was 30–37.4% (16). Compared with neurotypical children, children with ASD were at least three times more likely to suffer from concurrent GI symptoms, including constipation, diarrhea, abdominal pain and flatulence (17, 18). Clinical trials have only been conducted on children with ASD accompanied by GI symptoms (19, 20), but more data is needed to validate the efficacy of FMT in this population. The objective of this study is to investigate the utilization of FMT in children with ASD, both with and without GI symptoms, evaluate the clinical efficacy of FMT and analyze the alterations in bacterial and fungal composition within the gut microbiota. Furthermore, the study seeks to explore the mechanism underlying the improvement of ASD through FMT, focusing on the microbiota-gut-brain axis.

## Participants and methods

### Study design

**Figure 1:**
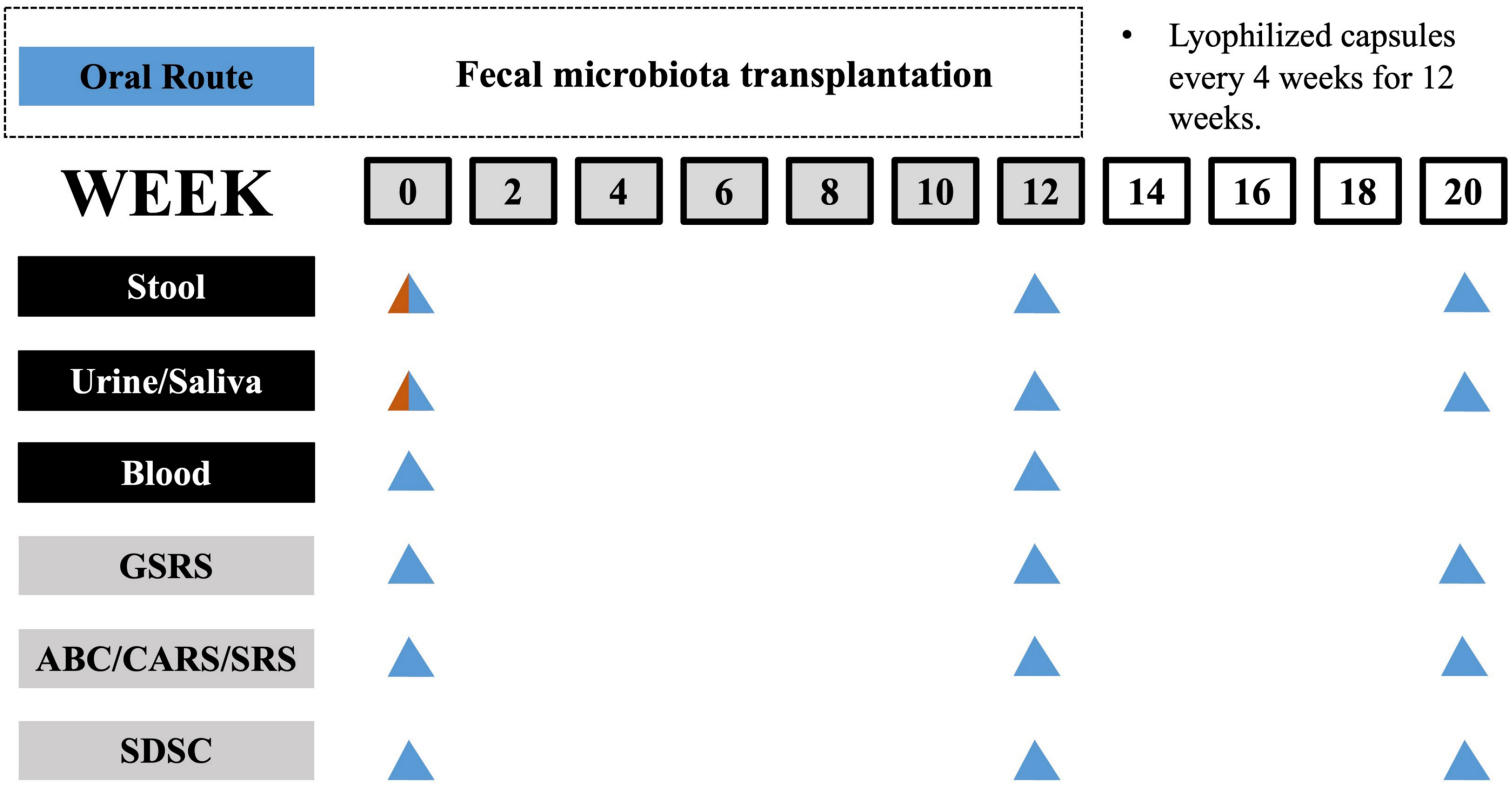
Timeline of study design. The study involves a 12-week treatment period for FMT followed by an 8-week observation period after FMT has ended. Sample collection and GI, behavior and sleep assessments were carried out at specific intervals, as indicated in a schematic timeline (bottom; healthy control and ASD group colored in red and blue, respectively).

As for sample size calculation, the estimated proportion of participants effectively treated with FMT is 90% (19). Using PASS 11 software, with *α* = 0.05 (two-sided) and power = 0.90, the calculated sample size is 35. Assuming a dropout rate of 10% for the study participants, a sample size of 38 is required. Finally, this study included 38 children with ASD (age 3–14 years) who were diagnosed by the criteria from the Diagnostic and Statistical Manual of Mental Disorders, Fifth Edition (DSM-5) by the American Psychiatric Association, and was conducted as an open-label clinical trial. Among these children with ASD, 31 children had GI symptoms (such as abdominal pain, reflux, indigestion, diarrhea and constipation) and 7 children had no GI symptoms. During the study, every child received a 12-week FMT treatment, followed by a 8-week observation period after the treatment ended. The entire study period lasted for 20 weeks. To serve as a healthy control (HC) group, 30 healthy children without GI symptoms were recruited. The healthy children were not treated and the FMT treatment was administered orally to all children with ASD via lyophilized capsules. Prior to FMT, neither vancomycin nor proton pump inhibitors were administered to any of the children. The researchers were aware of the group allocation and outcome evaluation. The study design is illustrated (Figure 1).

This clinical study was approved by the Ethics Review Committee at the Shanghai Children's Hospital, Shanghai Jiao Tong University. Before enrolling in the study, written informed consent was obtained from the guardian of each participant. Furthermore, the study was registered at the Chinese Clinical Trial Registry (www.chictr.org.cn). The trial protocol is accessible through the registration number ChiCTR2200055943.

### Subject recruitment

Children with ASD were primarily recruited from patients at the FMT multidisciplinary clinic of the Shanghai Children's Hospital. Healthy children were recruited from families of acquaintances of the study researchers. The inclusion criteria for the subjects include being under 18 years old and able to swallow capsules; meeting the diagnostic criteria for ASD in DSM-5 and commonly used diagnostic scales; having a legal guardian who fully understands and signs the informed consent form, agreeing to the researchers collecting clinical data and samples; and being able to participate in the study continuously and follow-up. Exclusion criteria included a definite diagnosis of Reye's syndrome or other mental illness; combined with a clear history of brain trauma, cerebral palsy, encephalitis and other organic brain diseases; the use of probiotics, antibiotics, proton pump inhibitors or other medications that affect the gut microbiota in the past 3 months; the presence of other serious GI diseases or organic lesions in the intestine such as congenital megacolon, intestinal obstruction, or intussusception; and poor compliance. The healthy children did not have any mental disorders, such as ASD, attention-deficit hyperactivity disorder, depression or anxiety, and none of them had a first-degree relative with ASD. Samples of stool, urine, saliva and blood were collected from children with ASD, while only initial samples of stool, urine and saliva were collected from healthy children. All participants' guardians were capable of comprehending and consenting to the informed consent, and all participants were capable of receiving follow-up evaluations for 8 weeks.

### Selection of stool donor and FMT preparation

The medical histories of the volunteers who underwent stool donor screening were reviewed and the laboratory test results were gathered. To be included as stool donors, individuals had to meet the following criteria: age between 15 and 30 years; overall good health; body mass index (BMI) ranging from 18.5 kg/m^2^ to 23.9 kg/m^2^; and regular bowel movements occurring once or twice per day with a Bristol stool score of type 4. Exclusion criteria included a previous history of GI or chronic systemic disease, malignant neoplasm or had received radiochemotherapy; combined with any ongoing diseases; the use of antibiotics, probiotics, prebiotics, proton pump inhibitors, immunosuppressant agents or blood products during the past 3 months; positive serum results for hepatitis A, B, or C, human immunodeficiency virus, syphilis, Epstein-Barr virus and cytomegalovirus; positive results of feces testing for bacteria, viruses and parasites, and detection of multidrug-resistant genes such as extended-spectrum beta-lactamases, carbapenemases and resistance to colistin (21). We recruited two donors in our study after screening.

A disposable bottle was employed to collect the fresh donor stool samples. 100 g of feces was mixed with 500 ml of saline to achieve homogeneity within a timeframe of 6 h, using automated microfiltration equipment (GenFMTer, Nanjing, Jiangsu Province, China). Afterwards, the fecal suspension was processed for filtration within the equipment according to the predetermined schedule (22). Following microfiltration, the suspension was gathered into 50 ml tubes and centrifuged at 1,500 g for 3 min. The supernatant was removed and the precipitate was dissolved in normal saline to form fecal bacterial solution. The lyophilized protective agent was added to the fecal bacterial solution, which was then freeze-dried into powder by low temperature freeze-drying machine. The ultimate lyophilized powder was double-encapsulated in size 00 hypromellose capsules (Anhui Huangshan Capsule, Xuancheng, Anhui Province, China) and kept at a temperature of −80°C for storage. The fecal capsules were transferred to a temperature of −20°C approximately 2 h before the oral administration.

The dosage and treatment duration of FMT for ASD are still under research, with variations in dosage and duration reported in previous literatures. The FMT dosages of each course were determined based on the weight of the donor stool and the body weight of the ASD children, with a ratio of 1 g of donor stool per 1 kg of recipient body weight. Based on our research on FMT treatment for pediatric diseases, the dosage of FMT is generally in line with the current ratio and has shown efficacy. Additionally, through the analysis of the dynamic changes in patients before and after FMT, the duration of microbiota transplantation is approximately 4 weeks*.* Therefore, we chose to use 3 treatment courses to enhance the colonization of gut microbiota (23–25). Each course of capsules were taken on an empty stomach before meals, completed within 3 days, with a frequency of one course every 4 weeks, totaling 3 courses over 12 weeks.

### Assessments and sample collection

Participants with ASD underwent the physical examination at week 0 of the study. Samples of stool were collected from ASD participants during week 0, 12 and 20 of the study for the purpose of analyzing the composition and abundance of gut microbiota through 16s rRNA and fungal ITS sequencing. Urine and saliva samples were collected at week 0, 12 and 20. Blood samples were collected at week 0 and 12. Urine and blood samples were collected for subsequent research on the mechanisms related to neurotransmitter metabolism and saliva samples for subsequent oral microbiota detection. Stool samples were collected from healthy children at the beginning of the study for 16s rRNA sequencing. The collected samples were processed immediately and stored at −80°C. After the FMT treatment, each participant was interviewed by phone. If any patients had questions or experienced adverse symptoms following the treatment, additional interviews or consultations would be conducted.

### Evaluations of GI symptoms

In order to evaluate the gastrointestinal symptoms of each participant, the parents were requested to fill out the Gastrointestinal Symptoms Rating Scale (GSRS). The GSRS is a self-reported questionnaire that uses a 7-point Likert scale with the following descriptive indicators: 1 (asymptomatic), 2 (slight), 3 (mild), 4 (moderate), 5 (moderate to severe), 6 (severe), and 7 (very severe) to assess the severity of gastrointestinal symptoms. It was initially designed as a hierarchical scale for face-to-face questioning to evaluate common gastrointestinal symptoms. It was later modified to create a self-management questionnaire. The GSRS is a 15-item questionnaire that assesses 5 dimensions of GI symptoms (abdominal pain, reflux, indigestion, diarrhea and constipation) experienced by participants in the last 2 weeks. The score for each dimension is calculated as the average score of all items within the dimension, ranging from 1 to 7.

### Evaluations of ASD related and sleep symptoms

To evaluate symptoms related to ASD, the following three scales were used: Autism Behavior Checklist (ABC), Childhood Autism Rating Scale (CARS) and Social Responsiveness Scale (SRS). The ABC includes 57 items that evaluates problem behaviors in five areas that are frequently observed in children with ASD. These areas are sensory, relating, body and object use, language, social and self-help. The CARS is a 15-item scale that can be utilized for both diagnosing ASD and evaluating the overall severity of the symptoms. The SRS is a 65-item questionnaire completed by parents or caregivers, and it assesses social communication, social cognition, social motivation and autistic mannerisms. The Sleep Disturbance Scale for Children (SDSC) is a questionnaire used to assess sleep problems in children. The SDSC consists of 26 items and evaluates six different domains of sleep disturbance. The ABC, CARS, SRS and SDSC were all given to participants at three different time points: baseline (week 0), 4 weeks after treatment (week 12) and at the final time of the evaluation period (week 20).

### Microbial DNA extraction

Microbial DNA was extracted from fecal samples by using the QIAamp DNA Stool Mini Kit (Qiagen, Hilden, Germany). Bacterial 16S rRNA V3-V4 hypervariable regions were amplified using polymerase chain reaction (PCR) primers 338F (5′-ACTCCTACGGGAGGCAGCAG-3′) and 806R (5′-GGACTACHVGGGTWTCTAAT-3′). Fungal internal transcribed spacer (ITS) regions were amplified using primers ITS1F (5′-CTTGGTCATTTAGAGGAAGTAA-3′) and ITS2R (5′-GCTGCGTTCTTCATCGATGC-3′). The PCR products were purified by using the AxyPrep DNA Gel Extraction Kit (Axygen Biosciences, Union City, CA, USA). Paired-end sequencing of purified amplicons were performed on an Illumina MiSeq PE300 platform (Illumina, Inc., San Diego, CA, USA) at Majorbio Bio-Pharm Technology Co., Ltd. (Shanghai, China).

### 16S rRNA and fungal ITS sequencing

FLASH (v1.2.11) was utilized to merge the raw 16S rRNA and ITS gene sequences and quality filtered with fastp (0.19.6) (26, 27). The high-quality sequences were then denoised using DADA2 to obtain unique Amplicon Sequence Variant (ASV) (28). The Qiime2 (version 2020.2) pipeline with recommended parameters was used for further analysis. Taxonomic assignment of ASV was conducted by using the Blast consensus taxonomy classifier implemented in Qiime2, the SILVA 16S rRNA database (v138) and the UNITE v8 database (29). The bacterial and fungal community composition data was analyzed by using the Majorbio Cloud Platform. The alpha diversity of the fecal microbiota was assessed using the observed richness (Sobs), Chao, abundance-based coverage estimator (ACE), Shannon and Simpson indexes. Moreover, the beta diversity was measured through principal coordinate analysis (PCoA) to Bray-Curtis distance and weighted UniFrac metric. The Wilcoxon rank-sum test was utilized to analyze the taxa difference in relative abundance between different groups.

### Statistical methods

In this study, the statistical analysis was conducted using SPSS 22.0 statistical software (SPSS Inc., Chicago, IL, USA). Continuous variables were represented as median and interquartile range (IQR). Normality tests were performed to guide the selection of statistical tests. The measurement data were compared using *t*-test, nonparametric Mann-Whitney *U*-test, Kruskal-Wallis *H*-test and Friedman test. In addition, the categorical data were compared using the chi-square test. All *p*-values were corrected with FDR, and if not stated otherwise, *p*-value less than 0.05 is regarded as statistically significant.

## Results

### Characteristics of ASD children

This study included 38 children who met the critical inclusion criteria for ASD, as well as 30 healthy children from separate families. The children with ASD in the study underwent FMT treatment for 12 consecutive weeks and were then monitored for an additional 8 weeks. The healthy children did not receive any treatment during the entire trial period (Figure 1). At the beginning of the trial, all participants were compared in terms of their demographic characteristics, such as gender, age distribution and body mass index (BMI) (Table 1). In addition, we assessed the severity of autism and GI symptoms in the children with ASD. Dietary assessment revealed that 47.4% (18 of 38) of ASD children had varying degrees of imbalanced dietary intake.

**Table 1:** Demographic characteristics of children with autism spectrum disorder (ASD) and healthy control (HC).

Characteristics	Healthy control (*n* = 30)	ASD children (*n* = 38)	*P*-value
Gender, male, *n* (%)	14 (46.7)	32 (84.2)	0.001
Age, years [median (IQR)]	6.5 (5.0–11.0)	7.0 (5.0–9.0)	0.58
Age range			
2–3 years old (*n*)	2	3	
4–6 years old (*n*)	13	10	
7–11 years old (*n*)	10	22	
12–17 years old (*n*)	5	3	
BMI, kg/m^2^ [median (IQR)]	18.1 (17.0–20.2)	16.7 (14.9–19.8)	0.09
Autism severity, *n* (%)			
Mild and moderate	NA	26 (68.4)	
Severe	NA	12 (31.6)	
Gastrointestinal symptoms, *n* (%)			
No	NA	7 (18.4)	
Abdominal pain	NA	6 (15.8)	
Reflux	NA	2 (5.3)	
Indigestion	NA	26 (68.4)	
Diarrhea	NA	15 (40.0)	
Constipation	NA	21 (55.3)	

IQR, interquartile range; BMI, body mass index; NA, not applicable.

*P*-value was calculated by the nonparametric Mann-Whitney test and comparisons of categorical data were performed by chi-square test.

### Clinical response to FMT

**Figure 2:**
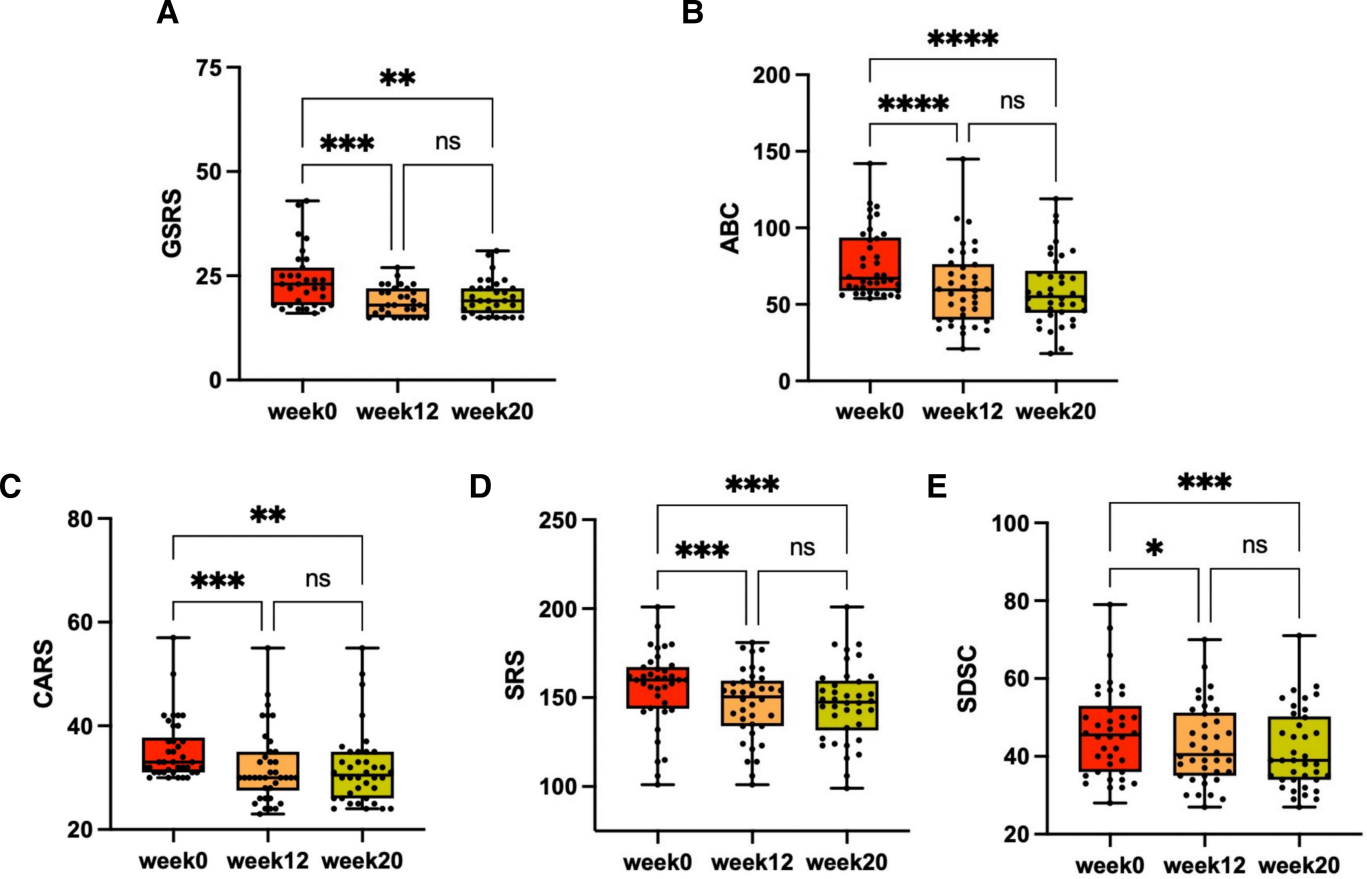
The changes in GI, ASD related and sleep symptoms after FMT. Every child with ASD received a 12-week FMT treatment, followed by an 8-week observation period after the treatment ended. GI, ASD related and sleep symptoms were assessed through scales at three different time points: baseline (week 0), after treatment (week 12) and at the final time of the evaluation period (week 20). (**A**) GSRS scores (*n* = 31). (**B**) ABC scores (*n* = 38). (**C**) CARS scores (*n* = 38). (**D**) Total SRS scores (*n* = 38). (**E**) SDSC scores (*n* = 38). The Friedman test was used to determine the significance. ns, no significance. *P < 0.05, **P < 0.01, ***P < 0.001, ****P < 0.0001.

In the follow-up phase, we used the GSRS to measure the overall GI symptoms of participants after FMT, and found significant improvement in symptoms such as indigestion, constipation, and diarrhea. The average GSRS score decreased by 51% after the cessation of FMT treatment but reversed (32% decrease from baseline) 8 weeks after treatment stopped (Figure 2A), indicating that GI symptoms were obviously improved after FMT. We also observed improvements in symptoms related to ASD and sleep after FMT treatment. After stopping FMT treatment, the ABC scores decreased by 20% and remained decreased (23% decrease from baseline) after 8 weeks of stopping treatment (Figure 2B). At the end of the follow-up phase, there was a 10% reduction in scores on the CARS (Figure 2C) and a 6% reduction in scores on the SRS (Figure 2D). After 8 weeks of stopping treatment, scores on the SDSC, which evaluates sleep symptoms, decreased by 10% (Figure 2E). Changes in each scale scores before and after FMT treatment were measured (Table 2). There were no dropouts during the trial. In addition, short-term adverse events observed during follow-up included vomiting and fever in 2 participants, which were self-limiting and resolved within 24 h, and no long-term adverse events were observed, indicating that FMT therapy is generally safe. Therefore, our data suggests that oral lyophilized FMT may improve GI, ASD related and sleep symptoms without causing any serious adverse events.

**Table 2:** Effect of FMT treatment on the GI, ASD related and sleep symptoms of ASD children.

	Week0		Week12		Week20
GSRS score [median (IQR)]	23.00 (18.00–27.00)		18.00 (15.00–22.00)		19.00 (16.00–22.00)
*P*-value		0.0002		1.000	
ABC score [median (IQR)]	67.00 (58.75–93.75)		59.50 (40.00–76.25)		55.00 (44.50–72.00)
*P*-value		<0.0001		1.000	
CARS score [median (IQR)]	33.00 (31.00–37.75)		30.00 (27.50–35.00)		30.50 (26.00–35.00)
*P*-value		0.0009		1.000	
SRS score [median (IQR)]	160.0 (143.8–167.3)		150.5 (134.0–159.5)		147.5 (131.5–159.5)
*P*-value		0.0003		1.000	
SDSC score [median (IQR)]	45.50 (36.00–53.00)		40.50 (35.00–51.25)		39.00 (34.00–50.25)
*P*-value		0.0103		0.906	

IQR, interquartile range.

*P*-value was calculated by the Friedman test.

### Altered gut bacterial microbiota in ASD children and changes after FMT

**Figure 3:**
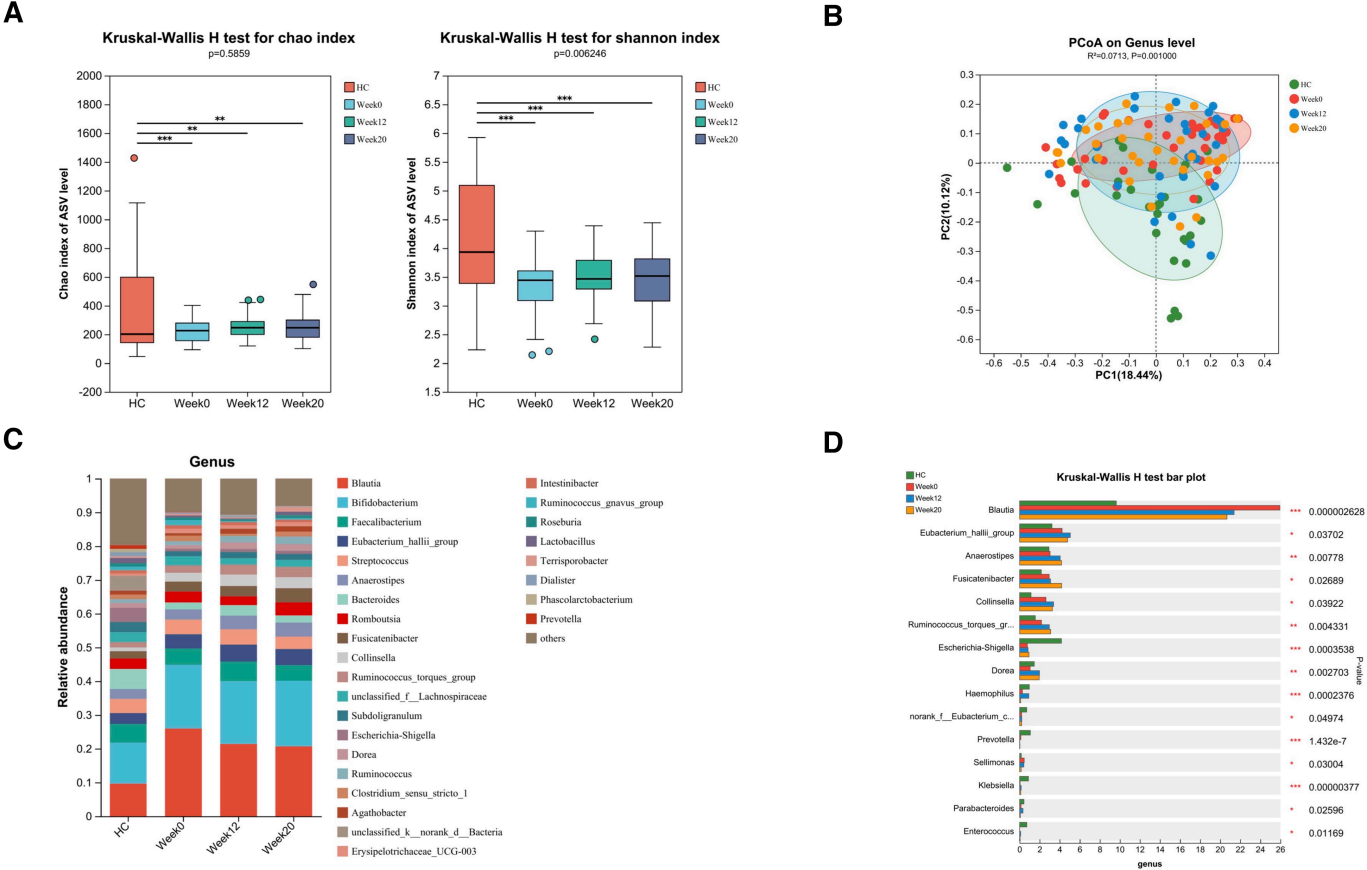
Altered gut bacterial microbiota in ASD children and changes after FMT. FMT treatment alters the composition of gut bacterial microbiota in children with ASD. (**A**) The α diversity measured by the Chao and Shannon indexes based on the ASV (HC, *n* = 30; ASD before FMT [week0], *n* = 38; After FMT [week12], *n* = 33; 8 weeks after FMT [week20], *n* = 32). Significance was determined by using Kruskal-Wallis *H*-test. (**B**) The β diversity analyzed by PCoA of Bray-Curtis distance (HC, *n* = 30; week0, *n* = 38; week12, *n* = 33; week20, *n* = 32). (**C**) The barplot showing the relative abundance of the representative genera in each group. (**D**) Bacterial genera abundances with significant differences. Significance was determined by using Kruskal-Wallis *H*-test. *P < 0.05, **P < 0.01, and ***P < 0.001.

**Figure 4:**
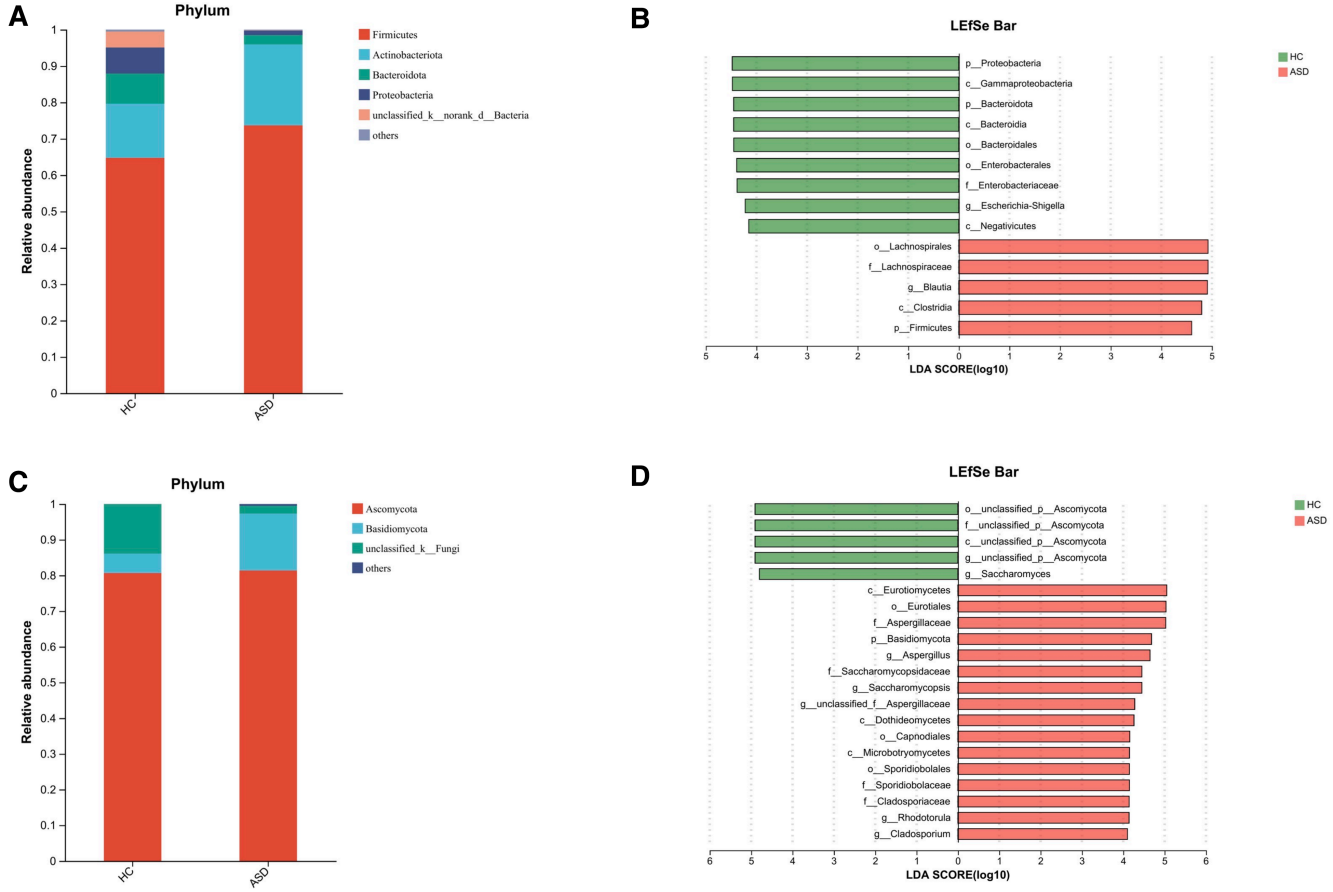
Altered gut microbiota in ASD children. The composition of gut microbiota in ASD children is different from that of HC. (**A**) The barplot showing the relative abundance of the most prevalent phyla of bacteria for the two groups. (**B**) LEfSe detected the taxa with the most significant differences in abundance of bacteria between the two groups. Bacteria with an LDA score greater than 4 were graphed. (**C**) The barplot showing the relative abundance of the most prevalent fungal phyla in the two groups. (**D**) LEfSe detected the taxa with the most significant differences in abundance of fungi between the two groups. Fungi with an LDA score greater than 4 were graphed.

We conducted an evaluation of alpha diversity and observed notable variations in alpha diversity between the HC and ASD groups during the initial assessment (Figure 3A). In order to investigate the dissimilarities in the gut microbiota of both groups, we computed beta diversity and detected significant disparities in beta diversity between the two groups (Figure 3B). When examining the microbiota composition at the phylum and genus levels, we noticed that there were dissimilarities between the microbiota of ASD patients and that of HC individuals (Figure 4A, Figure 3C). We used LEfSe analysis to identify two genera that showed significant differences in abundance between the ASD and HC group at baseline (LDA score greater than 4.0). Children in ASD group had a relatively higher abundance of *Lachnospiraceae* in family level, and *Blautia* in genus level, while HC group had a relatively higher abundance of *Enterobacteriaceae* in family level, and *Escherichia-Shigella* in genus level (Figure 4B).

After FMT treatment for 12 weeks, although there was no significant difference in alpha and beta diversity in children with ASD before and after FMT therapy (Figures 3A,B), FMT treatment resulted in alterations in the relative abundances of various bacterial genera in the samples of ASD patients. Specifically, there was an increase in the abundance of *Eubacterium_hallii_group, Anaerostipes, Fusicatenibacter, Collinsella*, *Ruminococcus_torques_group* and *Dorea*, and a decrease in the abundance of *Blautia*, *Prevotella* and *Sellimonas* (Figures 3C,D). The gut bacterial microbiota in ASD children with or without GI symptoms and changes after FMT were also analyzed, and we found that there may be significantly fewer individuals without GI symptoms, leading to no statistically significant differences in α and β diversity compared to the HC group (Supplementary Figures S1A,B). However, there are still differences in the abundance of specific bacterial genera (Supplementary Figures S1C,D).

### Fecal fungal microbiota changes after FMT

**Figure 5:**
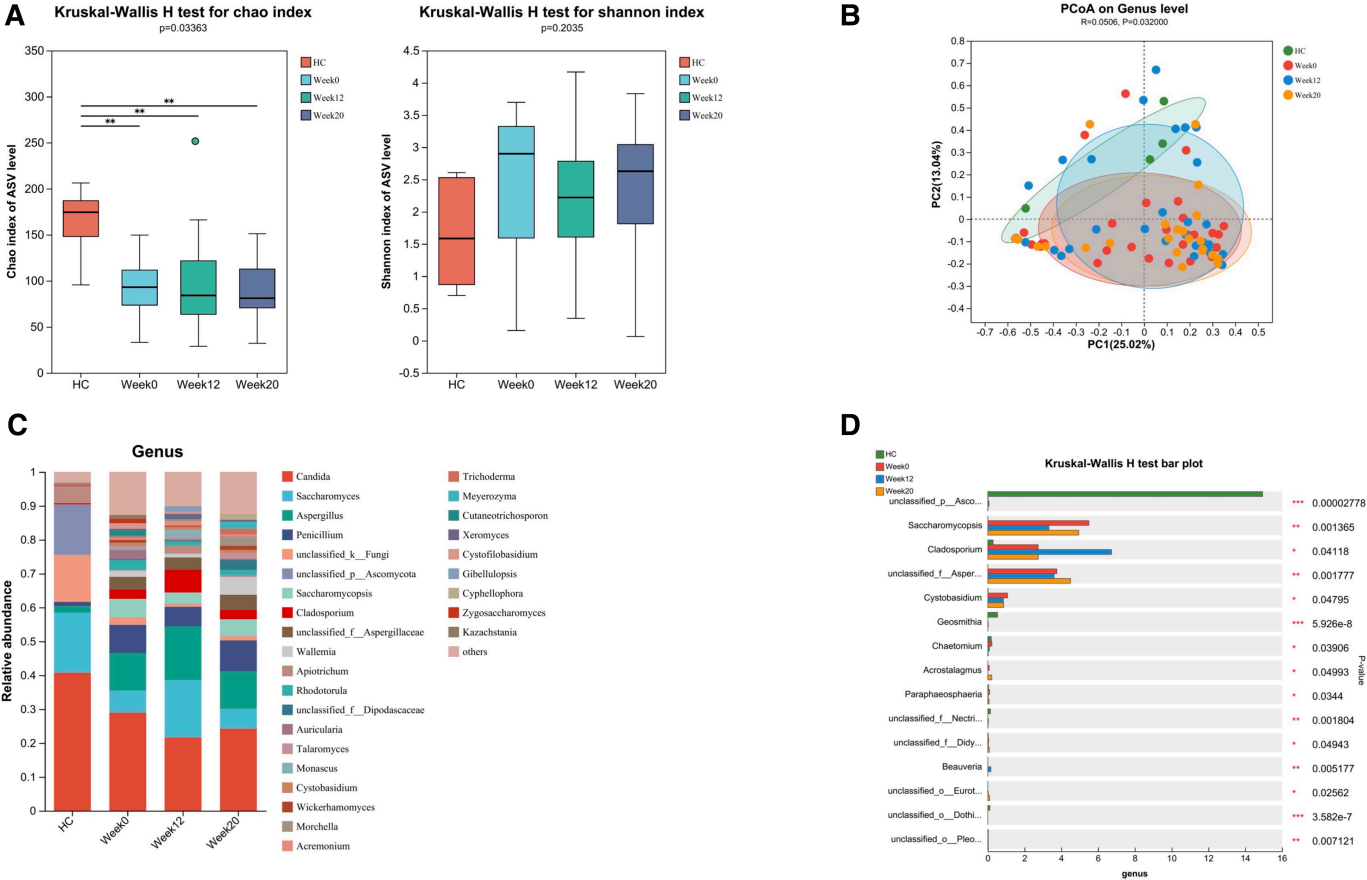
Altered gut fungal microbiota in ASD children and changes after FMT. FMT treatment alters the composition of gut fungal microbiota in children with ASD. (**A**) The α diversity measured by the Chao and Shannon indexes based on the ASV (HC, *n* = 5; ASD before FMT [week0], *n* = 31; 4 weeks after FMT [week12], *n* = 27; 12 weeks after FMT [week20], *n* = 23). Significance was determined by using Kruskal-Wallis *H*-test. (**B**) The β diversity analyzed by PCoA of Bray-Curtis distance (HC, *n* = 5; week0, *n* = 31; week12, *n* = 27; week20, *n* = 23). (**C**) The barplot showing the relative abundance of the representative genera in each group. (**D**) Fungal genera abundances with significant differences. Significance was determined by using Kruskal-Wallis *H*-test. *P < 0.05, **P < 0.01, and ***P < 0.001.

Through amplicon-based sequencing of the fungal ITS region, we analyzed the gut mycobiota of our study cohort. We found high-quality fungal sequences in 31 out of 38 individuals with ASD and in 5 out of 30 HC. We observed a significant difference in fungal alpha diversity between the HC and individuals with ASD (Figure 5A). The analysis of beta diversity using PCoA showed that the gut mycobiota of individuals with ASD differed significantly from that of the HC (Figure 5B). At both the phylum and genus levels, we observed differences in mycobiota composition between individuals with ASD and the HC (Figure 4C, Figure 5C). At baseline, we utilized LEfSe analysis to identify seven genera that exhibited significant differences in abundance between the ASD and HC (LDA score greater than 4.0). In terms of family-level abundance, children in the ASD group exhibited relatively higher levels of *Aspergillaceae, Saccharomycopsidaceae, Sporidiobolaceae* and *Cladosporiaceae*. At the genus level, ASD children had relatively higher levels of *Aspergillus, Saccharomycopsis*, *Rhodotorula* and *Cladosporium*. In contrast, the HC group had relatively higher levels of *Ascomycota* and *Saccharomyces* at the genus level (Figure 4D).

Following 12 weeks of FMT treatment, we did not observe any significant differences in alpha and beta diversity between baseline and post-treatment samples from children with ASD (Figures 5A,B), the FMT treatment induced changes in the relative abundances of several fungal genera in samples from patients with ASD. Notably, we observed an increase in the abundance of *Aspergillaceae* and a decrease in the abundance of *Saccharomycopsis*, *Cystobasidium* and *Chaetomium* (Figures 5C,D). The analysis also included the gut fungal microbiota in ASD children with or without GI symptoms, as well as the changes after FMT. Due to the significantly lower number of individuals without GI symptoms, we observed no statistically significant differences in α and β diversity compared to the HC group (Supplementary Figures S2A,B). Nonetheless, distinct differences in the abundance of specific fungal genera were still present (Supplementary Figures S2C,D).

### Covariance between ASD-related gut bacteria, fungi and clinical symptoms

**Figure 6:**
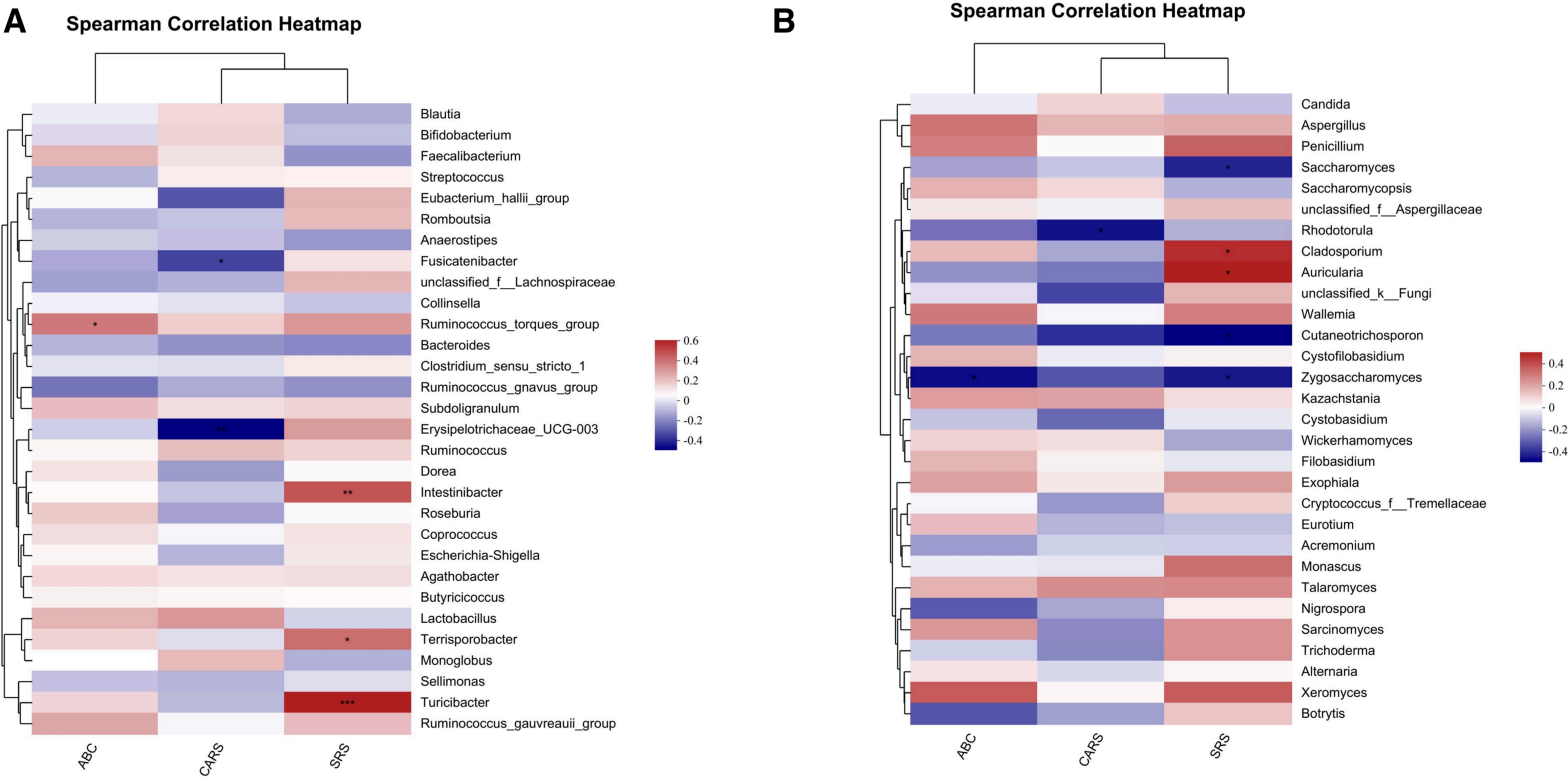
Covariance between ASD-related gut bacteria, fungi and clinical symptoms. Potential correlations of bacterial, fungal genera abundances and core symptoms among patients with ASD before and after FMT. (**A**) Spearman's correlation analysis between the top 30 significantly different gut bacterial genera and core symptoms of ASD. (**B**) Correlation matrices constructed from gut fungal genera and core symptoms of ASD by Spearman's correlation analysis. Positive and negative correlations are indicated as red and blue, respectively. *P < 0.05, **P < 0.01, and ***P < 0.001.

The associations of changed gut bacteria, fungi and clinical core symptoms in ASD patients were analyzed using Spearman's rank correlation coefficient. *Intestinibacter*, *Terrisporobacter* and *Turicibacter* were positively correlated with SRS score, including symptoms of social communication and cognition impairment. *Ruminococcus_torques_group* was positively correlated with ABC score, including five areas of sensory, relating, body and object use, language, social and self-help. On the other hand, increased abundances of *Fusicatenibacter* and *Erysipelotrichaceae_UCG-003* were negatively correlated with the scores of ASD core symptoms (Figure 6A). The increased abundances of certain fungal genera in patients with ASD, such as *Cladosporium* and *Auricularia,* were positively correlated with the scores of core symptoms. Conversely, increased abundances of *Saccharomyces, Rhodotorula, Cutaneotrichosporon* and *Zygosaccharomyces* were negatively correlated with the scores of ASD core symptoms (Figure 6B).

## Discussion

Few previous studies have explored the therapeutic effects of FMT on patients with ASD. In a small open-label clinical trial, Eighteen ASD children with moderate to severe GI symptoms underwent a 2-week course of antibiotics and bowel cleansing followed by FMT treatment, randomized to receive either orally or rectally, starting with a higher initial dose and then maintaining a lower dose for 7–8 weeks. Follow-up assessments were conducted 8 weeks after the treatment concluded. The treatment resulted in an 80% improvement in GI symptoms according to GSRS scores, and a 22% reduction in CARS scores during the treatment, followed by 24% after 8 weeks without treatment. Sequencing analysis showed that the abundance of *Bifidobacteria, Prevorella* and *Dephosphococcus*, as well as the total bacterial diversity, increased and remained stable after 8 weeks of treatment (19). Eight children with ASD showed improvements in both GI symptoms and ASD symptoms after 2 years of FMT treatment. Moreover, they experienced notable alterations in their gut microbiota, such as an increase in microbial diversity and a higher proportion of *Bifidobacteria* and *Prevotella* (30). The above clinical trial included a small number of ASD children, all with moderate to severe GI symptoms. The FMT treatment methods varied, with children requiring daily oral or rectal administration during the treatment period. Our study included more ASD children and utilized freeze-dried powder capsules for oral administration, reducing the frequency of dosing. This approach helps alleviate medication challenges for many ASD children and parental anxiety.

Another clinical trial involved 40 children with ASD and 16 healthy children who underwent 4 weeks of FMT treatment followed by an 8-week follow-up, with bowel cleansing performed before the treatment. The results indicated that both oral and rectal FMT were effective, and FMT also promoted the engraftment of the donor microbiota. There was a positive correlation between the abundance of *Eubacterium coprostanoligenes* and high GSRS scores, but FMT treatment decreased its abundance, which may have contributed to the improvement of ASD symptoms (20). The ASD children included in the above study all had GI symptoms. The treatment effectiveness for ASD children without GI symptoms is unknown and the treatment duration was relatively short. The results suggested that targeting specific microbial interventions may enhance the effectiveness of FMT, but further in-depth research is needed. This could provide valuable insights for optimizing treatment strategies. Our study included ASD children without GI symptoms, and we conducted correlation analysis between the changes in gut bacterial and fungal composition before and after treatment and the improvement in core ASD symptoms such as social impairment. These findings suggest that FMT may be a safe and effective long-term therapeutic option for improving ASD symptoms by modulating the gut microbiota.

The etiology of ASD remains unclear, but there is a certain correlation between gut bacterial community and ASD. The microbiota in children with ASD showed a relatively stagnant development and gradually deviated from the normal track. In addition, the initial microbiota was unstable and underdeveloped, and it was challenging for common bacteria to establish colonization. The relationship of the microbiome significantly changed before the age of 3 in children with ASD, which is consistent with the age at which behavioral deficits occur (31). Multiple studies have shown that the gut bacterial community in ASD patients is consistently different from that in HC (32). In comparison to healthy children, children with ASD displayed a reduction in the abundance of *Verrucomicrobia* at the phylum level (33). *Betaproteobacteria* increased at the class level, *Clostridiales* increased at the order level, *Bacteroidales* and *Selenomonadales* decreased at the order level (34). From the family level, *Erysipelotrichaceae* and *Ruminococcaceae* had higher levels, and *Prevotellaceae, Actinomycetaceae, Coriobacteriaceae, Streptococcaceae, Bifidobacteriaceae* and *Oscillospira* had lower levels in children with ASD (35). *Clostridium, Alkaliflexus, Desulfovibrio, Acetanaerobacterium, Parabacteroides, Lactobacillus, Sutterella, Odoribacter, Butyricimonas, Prevotella, Dorea, Collinsella, Lachnoclostridium, Bifidobacterium, Coprobacillus* elevated in ASD children, while *Weissella, Helcococcus, Alkaliphilus, Anaerofilum, Pseudoramibacter, Streptococcus, Anaerovorax, Lactococcus, Leuconostoc, Ethanoligenens, Veillonella, Flavonifractor, Haemophilus, Eisenbergiella, Akkermansia, Faecalibacterium, Parasutterella, Paraprevotella* reduced in the genus level (8, 36, 37). These findings regarding changes in microbiota in each study may vary due to differences in sample size and population. In our study, we found that *Blautia* and *Sellimonas* were increased at the genus level in children with ASD compared to HC, while *Dorea* was decreased. However, after FMT treatment, *Blautia* and *Sellimonas* decreased while *Dorea* increased. Moreover, the increased abundances of *Fusicatenibacter and Erysipelotrichaceae_UCG-003* were negatively correlated with the scores of ASD core symptoms.

There are some studies that have explored the relationship between ASD and gut fungi. Some of these studies have found that dysbiosis of gut fungi can affect the development of ASD. Candida is a prevalent and widely distributed genus within the human gut mycobiota (38). *Candida albicans* is significantly increased in the gut of individuals with ASD. The expansion of *Candida* in the gut mycobiota of individuals with ASD may lead to alterations in the intestinal fungal population, which could potentially have negative effects on GI abnormalities through cytokine dysregulation (39). Another study revealed a high prevalence of GI yeast in individuals with ASD, with *Candida* detected in 57.5% of the ASD group but not in the control group using a simple culture-based method. Additionally, the identification of aggressive forms of *Candida* (pseudo-hyphae presenting) under a light microscope suggests that these yeasts may more easily adhere to the intestinal mucosa (40). To our knowledge, there are currently no studies linking alterations in gut mycobiota with FMT therapy for ASD. In our study, we found that *Saccharomycopsis* and *Cystobasidium* were increased at the genus level in children with ASD compared to HC. However, after FMT treatment, *Saccharomycopsis* and *Cystobasidium* decreased. Children with ASD did not have a higher abundance of *Candida* compared to HC, but the abundance of *Candida* decreased after FMT treatment. Moreover, the increased abundances of *Saccharomyces, Rhodotorula, Cutaneotrichosporon* and *Zygosaccharomyces* were negatively correlated with the scores of ASD core symptoms.

There are some limitations to our study. The open-label design, absence of a placebo group, recruitment biasa nd the limitations of 16S rRNA sequencing for strain-specific analysis are critical considerations when interpreting the study results. Firstly, our study was a small, single-center intervention study. It is possible that the observed impact on ASD and GI symptoms may be influenced by placebo effects. Larger randomized controlled trials are needed to further investigate the efficacy of FMT in children with ASD. Secondly, we assessed the clinical response to FMT only at 2 months. Since there is a possibility of symptom relapse, it is important to conduct long-term follow-up of patients. Thirdly, the number of participants without GI symptoms at the time of enrollment was limited, and the limited dietary patterns common in children with ASD could potentially influence the composition of gut microbiota. Finally, our analysis of the gut microbiota in children with ASD was limited to fecal bacteria and fungi in the short term, we need to conduct long-term studies that focus on the microbiota in various parts of the GI tract to gain a better understanding of how FMT affects the gut microbiota of children with ASD.

## Conclusion

In summary, our study found that oral administration of lyophilized FMT is an effective and safe treatment for children with ASD. FMT could improve GI, ASD related symptoms, as well as sleep disturbances, and alter the gut bacterial and fungal microbiota composition in children with ASD.

## Data Availability

Raw sequencing data have been deposited in the NCBI Sequence Read Archive database under study accession number PRJNA1047541.

## Supplementary material

Supplementary Figure S1 Altered gut bacterial microbiota in ASD children with or without GI symptoms and changes after FMT. Comparison of gut bacterial microbiota between ASD children with GI symptoms and without GI symptoms. (**A**) The α diversity of gut bacterial microbiota measured by the Chao and Shannon indexes based on the ASV (HC, *n* = 30; ASD with GI symptoms, *n* = 31; ASD without GI symptoms, *n* = 7). (**B**) The β diversity analyzed by PCoA of Bray-Curtis distance. (**C**) and (**D**) Bacterial genera abundances with significant differences. Significance was determined by using Kruskal-Wallis *H*-test. **P* < 0.05, ***P* < 0.01, and ****P* < 0.001.

Supplementary Figure S2 Altered gut fungal microbiota in ASD children with or without GI symptoms and changes after FMT. Comparison of gut fungal microbiota between ASD children with GI symptoms and without GI symptoms. (**A**) The α diversity of gut fungal microbiota measured by the Chao and Shannon indexes based on the ASV (HC, *n* = 5; ASD with GI symptoms, *n* = 26; ASD without GI symptoms, *n* = 5). (**B**) The β diversity analyzed by PCoA of Bray-Curtis distance (ASD with GI symptoms, *n* = 26; ASD without GI symptoms, *n* = 5). (**C**) and (**D**) Fungal genera abundances with significant differences. Significance was determined by using Kruskal-Wallis *H*-test. **P* < 0.05, ***P* < 0.01, and ****P* < 0.001.
